# Ayahuasca enhances the formation of hippocampal-dependent episodic memory without impacting false memory susceptibility in experienced ayahuasca users: An observational study

**DOI:** 10.1177/02698811241301216

**Published:** 2024-11-29

**Authors:** Manoj K Doss, Lilian Kloft, Natasha L Mason, Pablo Mallaroni, Johannes T Reckweg, Kim van Oorsouw, Nina Tupper, Henry Otgaar, Johannes G Ramaekers

**Affiliations:** 1 Department of Psychiatry and Behavioral Sciences, Center for Psychedelic Research & Therapy, The University of Texas at Austin Dell Medical School, Austin, TX, USA; 2 Department of Psychiatry and Behavioral Sciences, Center for Psychedelic & Consciousness Research, Johns Hopkins University School of Medicine, Baltimore, MD, USA; 3 Department of Neuropsychology and Psychopharmacology, Faculty of Psychology and Neuroscience, Maastricht University, Maastricht, Netherlands; 4 Department of Clinical Psychological Science, Faculty of Psychology and Neuroscience, Maastricht University, Maastricht, Netherlands; 5 Leuvens Institute of Criminology, Faculty of Law and Criminology, KU Leuven, Leuven, Belgium

**Keywords:** Episodic memory, false memory, recollection, familiarity, psychedelics, ayahuasca

## Abstract

**Background:**

Ayahuasca is an Amazonian brew with 5-HT_2A_-dependent psychedelic effects taken by religious groups globally. Recently, psychedelics have been shown to impair the formation of recollections (hippocampal-dependent episodic memory for specific details) and potentially distort memory while remembering. However, psychedelics spare or enhance the formation of familiarity-based memory (cortical-dependent feeling of knowing that a stimulus has been processed).

**Aims:**

Given the growing literature on the plasticity-promoting effects of psychedelics, we investigated the acute impact of ayahuasca on recollection, familiarity, and false memory in an observational study of 24 Santo Daime members with >500 lifetime ayahuasca uses on average.

**Methods:**

Participants completed a false memory task at baseline and after they consumed a self-selected dose of ayahuasca prepared by their church (average dose contained 3.36 and 170.64 mg of *N,N*-dimethyltryptamine and β-carbolines, respectively).

**Results:**

Surprisingly, pre-encoding administration of ayahuasca enhanced hit rates, memory accuracy, and recollection but had no impact on familiarity or false memory. Although practice effects cannot be discounted, these memory enhancements were large and selective, as multiple measures of false memory and metamemory did not improve across testing sessions. β-carboline activity potentially accounted for this recollection enhancement that diverges from past psychedelic research. Although ayahuasca did not impact familiarity, these estimates were generally elevated across conditions compared to past work, alluding to a consequence of frequently driving cortical plasticity.

**Conclusions:**

When encoding and retrieval took place under acute ayahuasca effects in experienced ayahuasca users, susceptibility to memory distortions did not increase, potentially owing to enhancements in memory accuracy.

## Introduction

Ayahuasca is a psychedelic Amazonian brew containing *N,N*-dimethyltryptamine (DMT) from *Psychotria viridis* and β-carbolines from *Banisteriopsis caapi*. Although DMT degrades rapidly, the β-carbolines allow it to be orally active by inhibiting monoamine oxidase A (MAO-A). Like psychedelics such as psilocybin and lysergic acid diethylamide (LSD), activation of 5-HT_2A_ receptors largely contributes to ayahuasca’s psychoactive effects (Valle et al., 2016), and DMT (D’Souza et al., 2022) and ayahuasca (Palhano-Fontes et al., 2019; Sanches et al., 2016) are showing promise for the treatment of depression. Nevertheless, DMT possesses a unique pharmacology including σ receptor activation that may induce hippocampal neurogenesis (Morales-Garcia et al., 2020) and therefore impact episodic memory. Moreover, whereas standard MAO-A inhibitors (e.g., moclobemide) do not produce substantial subjective effects, β-carbolines can be psychoactive on their own (Ott, 1999) and to some degree act as 5-HT_2A_ agonists (Glennon et al., 2000; Grella et al., 1998) and GABA_A_ negative allosteric modulators (Robertson, 1980; Rommelspacher et al., 1981). Ayahuasca is consumed by several religious groups who take it multiple times per month, and research on these groups can contribute to understanding the impact of long-term psychedelic use on neurocognitive processes (e.g., Bouso et al., 2012, 2015; Doering-Silveira et al., 2005; Grob et al., 1996).

Recently, there has been growing interest in the impact of psychedelics on episodic memory (Doss et al., 2023a, 2023b; Healy, 2021; Kloft et al., 2021), the conscious re-experiencing of information from the past. Episodic memory is thought to rely on hippocampal and cortical plasticity, and the therapeutic efficacy of psychedelics has been proposed to rely on their induction of plasticity, especially in the cortex (Calder and Hasler, 2023; Inserra et al., 2021; Olson, 2018). States of heightened plasticity may allow for the revision of maladaptive memories, though they may also provide fertile ground for the formation of memories for non-experienced details of events or false memories (Otgaar et al., 2021).

How ayahuasca may impact the formation (encoding), stabilization (consolidation), and remembering (retrieval) of memories is complicated by its polypharmacology. On the one hand, β-carbolines may enhance encoding, as increasing synaptic monoamines prior to the presentation of stimuli can enhance subsequent memory for these stimuli (e.g., dextroamphetamine, Ilieva et al., 2015), and the MAO-A inhibitor moclobemide can reverse memory impairments from depression, aging, and pre-encoding administration of scopolamine (Allain et al., 1992; Wesnes et al., 1989). However, pre-encoding psilocybin administration impairs memory (Barrett et al., 2018), suggesting that DMT should also impair encoding. Likewise, pre-encoding ±3,4-methylenedioxymethamphetamine (MDMA) administration impairs memory (Doss et al., 2018a; Kloft et al., 2022; van Wel et al., 2011) in a 5-HT_2A_-dependent fashion (van Wel et al., 2011), even though MDMA increases synaptic monoamines, suggesting that 5-HT_2A_ activation via MDMA’s *R*-enantiomer underlies its encoding impairment.

Note that pre-encoding drug effects persist after stimuli are encoded, thereby potentially impacting consolidation, and many studies have administered a drug prior to encoding and tested memory without a sobering delay, thereby also impacting retrieval (Doss and Das, 2024). To isolate drug effects to consolidation, some studies have administered drugs immediately post-encoding and tested memory after a ⩾24-h delay to preclude drug effects on retrieval. Whereas post-encoding increases in monoamines via dextromethamphetamine or methylphenidate administration did not impact memory using this design (Ballard et al., 2015; Wagner et al., 2017), post-encoding LSD administration enhanced memory (Wießner et al., 2022), consistent with animal work (Zhang et al., 2013). Although no study has isolated drug effects of a prototypical psychedelic to retrieval on an objective memory test (i.e., sober encoding, delay for consolidation, retrieval under drug effects), one study found subjective vividness ratings under psilocybin during autobiographical memory retrieval were enhanced (Carhart-Harris et al., 2012). Nevertheless, the veracity of these memories was not assessed (cf. Pernot-Marino et al., 2004), and various psychoactive drugs, including MDMA and dextroamphetamine during retrieval increase false memory (Doss et al., 2023a). Susceptibility to false memory formation may especially be true of psychedelics, which enhance mental imagery (de Araujo et al., 2012; Kraehenmann et al., 2017; but see Ramaekers et al., 2023) and primary suggestibility (Carhart-Harris et al., 2015), factors linked to increased false memory (Schacter, 1999; Weinstein and Shanks, 2010).

Another complexity regarding the effects of psychedelics on episodic memory is differential modulation of distinct memory processes, specifically, recollection and familiarity (Yonelinas, 2002). Recollection is the hippocampal-dependent retrieval of details involving self-specific information (e.g., when and where one experienced an event or idiosyncratic associations one made during encoding), whereas familiarity is the cortical-dependent feeling of knowing that a stimulus has been experienced without retrieving corroborating evidence (e.g., recognizing a face but not remembering how one knows this individual). Pre-encoding administration of GABA_A_ sedatives (e.g., alcohol), NMDA dissociatives (e.g., ketamine), and THC impairs recollection, as well as familiarity (Doss et al., 2023b). Like these other drugs and consistent with the expression of 5-HT_2A_ receptors on inhibitory neurons in the hippocampus and the input to the hippocampus (i.e., entorhinal cortex; Bombardi, 2012; de Filippo et al., 2021; Deng and Lei, 2008; Lüttgen et al., 2004; Shen and Andrade, 1998; Wyskiel and Andrade, 2016), pre-encoding psilocybin and MDMA administration impaired memory on a recollection-based task (verbal-free recall; Barrett et al., 2018; van Wel et al., 2011) and recollection estimates from the dual process signal detection (DPSD) model (Doss et al., 2023b). However, DPSD familiarity estimates tended to be enhanced by pre-encoding psilocybin and MDMA administration, perhaps owing to 5-HT_2A_ expression on excitatory cortical neurons (Aghajanian and Marek, 1997; Jakab and Goldman-Rakic, 1998; Martin and Nichols, 2016). Moreover, MDMA administered prior to the encoding of Deese-Roediger-McDermott lists (e.g., *bed, rest, awake*, etc.) was found to increase false alarms to related lures that may seem familiar (e.g., *tired*), whereas false alarms to critical lures (e.g., sleep; Kloft et al., 2022), which typically involve both recollection and familiarity (Gallo, 2010), were unimpacted (cf. Doss et al., 2024). Interestingly, non-pharmacological studies have found that when recollection fails and familiarity is high, phenomena sometimes reported under psychedelics can occur such as *déjà vu* (Cleary et al., 2012), *presque vu* (illusory feelings of insight, Kostic et al., 2015), and premonition (Cleary and Claxton, 2018). Together, these findings highlight the importance of delineating drug effects on memory phases, memory processes, and stimuli with varying degrees of overlapping content, as such effects can conspire to produce cognitive illusions (cf. Doss et al., 2020b).

In the present observational study, we examined how ayahuasca impacts true and false memory in members of the Santo Daime church, who have consumed ayahuasca multiple times per month for several years. While sober and under ayahuasca, participants completed an episodic memory task containing a misinformation phase between the encoding and retrieval phases aimed at distorting memory (cf. Loftus, 2005). How psychedelics could impact false memory when drug effects are present across these three phases is an important question, as a significant event can happen during acute effects that is soon followed by elaboration and querying one’s memory. Such a situation may be particularly relevant to frequent ayahuasca users who interact with each other under acute effects during ceremonies. In addition to measuring recollection, familiarity, and false memory, we measured metamemory, the capacity to understand one’s own memory (Fleming and Lau, 2014), as the etymology of “psychedelic,” being “mind revealing,” would suggest that psychedelics increase insight into one’s own cognitive processes. Although psychedelics have not been found to impact metacognition, increasing synaptic monoamines via dextroamphetamine and dextromethamphetamine during encoding or retrieval has been shown to enhance metamemory (Doss et al., 2023b).

## Methods

### Participants

Twenty-four Santo Daime members (14 males; see Table 1 for demographics) were enrolled, an *N* at least as large as similar pharmacological investigations of episodic memory (Doss et al., 2023b). Exclusion criteria comprised MRI contraindications, pregnancy (confirmed with urine tests), use of drugs in the past 24 h that interact with MAO-A inhibitors, use of a psychedelic (other than ayahuasca; note that 20 of the 24 participants had participated in a ceremony in the 3 weeks prior to testing) in the past 3 months, use of other psychoactive drugs in the past 7 days (confirmed with urine tests), and use of alcohol in the past 24 h (confirmed with breath tests). These participants were experienced ayahuasca users with a mean (standard deviation (SD)) membership duration of 14.2 (8.3) years, attendance of 563 (650) ceremonies (range: 50–2700), age of 55.2 (10.2) years, and weight of 75.5 (12.6) kg. All participants provided informed consent, and the study was conducted in accordance with the Declaration of Helsinki (1964) amended in Fortaleza (Brazil, October 2013) and with the Medical Research Involving Human Subjects Act (WMO) that was approved by the Academic Hospital and Maastricht University’s ethics committee.

**Table 1.** Demographics.

Variable	Mean ± SD or %
Sex (male)	14 (60.8%)
Age (years)	37.91 ± 10.31
Weight (kg)	75.24 ± 12.10
Height (m)	1.78 ± 0.06
BMI (kg/m²)	23.57 ± 3.32
Years in Santo Daime	14.43 ± 8.39
Number of ceremonies	566.73 ± 665.17
Days since the last ceremony	48.21 ± 96.20
Dose (mL)	24.30 ± 8.32
Dose/weight (mL/kg)	0.33 ± 0.11

SD: standard deviation.

### Study design and drug

This observational study used a within-subject, fixed-order design and is described in detail elsewhere (Mallaroni et al., 2024; Ramaekers et al., 2023). Participants completed measures while sober (baseline) and 24 h later under the acute effects of a self-selected dose of ayahuasca (*M* = 24 mL, SD = 8.16, range = 11–40 mL) consumed in a ceremonial context with other Santo Daime members who were also tested in this study (six members per ceremony). A single batch of ayahuasca was prepared by the Santo Daime church, and the research team was not involved in producing or administering the ayahuasca. Alkaloid concentrations in a sample were determined after dissolution in 25 mL of water using high-performance liquid chromatography coupled to mass spectrometry, which was calibrated with pure reference substances of DMT (Cerilliant, Round Rock, TX, USA), harmine, harmaline (Aldrich Chemistry, St. Louis, MO, USA), and tetrahydroharmine (LGC Standards, Wesel, Germany). This sample contained 0.14, 4.50, 0.51, and 2.10 mg/mL of DMT, harmine, harmaline, and tetrahydroharmine, respectively. Thus, the average doses of DMT and combined β-carbolines were 3.36 and 170.64 mg, respectively.

### False memory task

This task is based on a previous study with several changes (Moritz et al., 2006). During the baseline session, participants completed a practice version of the task before the full version, and during the ayahuasca session, they began the full version of the memory task ≈160 min post-ayahuasca ingestion. Participants completed other measures before and after the memory task that are reported elsewhere (Madrid-Gambin et al., 2022; Mallaroni et al., 2024; Ramaekers et al., 2023). The memory task consisted of three phases: encoding, misinformation, and retrieval. During each encoding phase, participants viewed three of six illustrated black and white scenes comparable in complexity (classroom, beach, funeral, surveillance room, ayahuasca ceremony, and ayahuasca brewing). Scenes were presented in randomized order each for 40 s and were not repeated between sessions. Participants were instructed to attend to details because their memory would be tested. After each scene, the name of the picture was displayed, and participants had to rate how familiar they were with the scene on a 0–100 scale. Scenes were counterbalanced across participants such that they occurred an equal number of times in baseline and ayahuasca sessions (stimuli and counterbalancing procedure can be found at https://doi.org/10.17605/OSF.IO/2WS5Y).

After the encoding phase, participants completed a 1-min filler task (Raven’s Progressive Matrices) (John and Raven, 2003) followed by the misinformation phase (cf. Loftus, 2005). Three narratives were presented each describing a scene from the encoding phase for ≈30 s. Participants were told that these narratives were from former participants describing the scenes from memory and that some information in these narratives may be inaccurate. Each narrative suggested four objects that were actually presented in the scene and four objects that were not presented in the scene but were semantically central to the scene (e.g., a beach towel suggested for the beach scene).

After the misinformation phase, participants completed another 1-min filler task (a different version of Raven’s Progressive Matrices) followed by the retrieval phase. The retrieval phase consisted of a self-paced cued recollection task containing 72 trials in randomized order. On each trial, participants were asked if they saw a particular object in one of the scenes (yes/no) followed by a confidence rating (4-point scale). Participants were told to only respond based on what they saw in the scenes and not based on the narratives of the misinformation phase. For each scene, there were eight objects presented in the scene and not suggested in the misinformation phase (targets), four objects presented in the scene and suggested in the misinformation phase (suggested targets), four objects not presented in the scene but suggested in the misinformation phase (suggested lures), four objects not presented in the scene or suggested in the misinformation phase but related to objects presented in the scene (related lures), and four objects not presented in the scene, suggested in the misinformation phase, or related to objects in the scene (unrelated lures). Thus, for each participant and drug condition, there were 24 targets, 12 suggested targets, 12 suggested lures, 12 related lures, and 12 unrelated lures. Due to a computer error, 12 participants in each drug condition were missing data for 1 target, and 6 participants in each drug condition were missing data for 1 unrelated lure.

### Data analysis

Pre-registered hypotheses and data can be found at https://doi.org/10.17605/OSF.IO/2WS5Y. We predicted that ayahuasca would impair (true) memory for stimuli that were presented (due to acute effects on encoding) and increase false alarms across suggested, related, and unrelated lures (due to acute effects during retrieval). We expected there to be a particularly robust increase in false alarms to suggested lures given that psychedelics can increase suggestibility and acute drug effects were present during the misinformation phase. Finally, we predicted that enhanced visual imagery would mediate these false alarms and that false alarms would be overall larger for ayahuasca-related stimuli given the familiarity of these contexts. However, false alarms were not increased (for any of the three types of lures), visual imagery was not enhanced (Ramaekers et al., 2023), and there were no differences in memory performance between ayahuasca and non-ayahuasca scenes. Thus, analyses were combined across scenes to minimize comparisons and increase power (cf. Adam et al., 2020). Memory performance for each drug condition was first analyzed for hit rates (*p*[“yes”|target]), suggested hit rates (*p*[“yes”|suggested target]), suggested lure false alarm rates (*p*[“yes”|suggested lure]), related lure false alarm rates (*p*[“yes”|related lure]), and unrelated lure false alarm rates (*p*[“yes”|unrelated lure]). We also explored the data using measures that controlled for response bias. A standard measure of memory accuracy comparable to memory paradigms without misinformation can be computed from the difference between hit rates and unrelated lure false alarm rates. A more fine-grained memory measure (“precision”) comparable to tasks containing related lures with a high incidence of false alarm rates (e.g., Doss et al., 2020b) can be computed from the difference between hit rates and related lure false alarm rates. Likewise, precision can be computed for items with suggested information (“suggested precision”) from the difference between suggested hit rates and suggested false alarm rates. To determine the effect of suggestion on false memory that controls for an item’s relatedness to stimuli from encoding (“suggestion”), related lure false alarm rates were subtracted from suggested lure false alarm rates. To determine the effect of relatedness on false memory that controls for a general response bias (“relatedness”), unrelated lure false alarm rates were subtracted from related lure false alarm rates. High-confidence versions of all these measures can be computed by using only “yes” responses given the highest level of confidence. To test the effects of ayahuasca on these measures and average familiarity ratings of the scenes during the encoding phase, repeated measures *t*-tests were conducted (α = 0.050). Exploratory correlations between all these measures and plasma concentrations of DMT, harmine, harmaline, tetrahydroharmine, and total β-carbolines, as well as amount of consumed ayahuasca (previously reported in Madrid-Gambin et al., 2022; Ramaekers et al., 2023) are reported in the Supplemental Material. Given the large number of tests, these correlations should be interpreted with caution.

Note that many of the memory performance metrics are related and could be influenced by the same latent processes. For example, an accurate recollection for the scenes could increase hit rates and decrease all types of false alarms, and high familiarity for scenes or misinformation could increase false alarms to related and suggested lures, respectively. Moreover, a good understanding of one’s memory or the task (especially after already performing the task once) could influence confidence ratings. Thus, confidence data were submitted to a DPSD analysis (Yonelinas, 1994, 2002) to estimate recollection and familiarity and a meta-*d*′ analysis to estimate metamemory (Fleming and Lau, 2014; Maniscalco and Lau, 2012).

The receiver operator characteristic (ROC) Toolbox for Matlab was used for DPSD modeling and is discussed in detail elsewhere (Koen et al., 2017). Briefly, hit rates are plotted against false alarm rates in a cumulative fashion starting with the highest level of confidence to the lowest level of confidence with the final point being (1,1). A ROC curve is then fit to these points using maximum likelihood estimation, but unlike a standard ROC curve, the *y*-intercept is allowed to vary. Recollection estimates come from this *y*-intercept, whereas familiarity estimates come from the curvilinearity of the function. Because the trial count per participant was low for model-fitting, we ran a previously used bootstrapping procedure (Doss et al., 2018a, 2018c, 2023b). For each drug condition, 24 participants (this study’s *N*) were sampled with replacement 10,000 times. On each iteration, confidence counts were collapsed across the sampled participants and fit with the DPSD model to generate distributions of recollection and familiarity estimates. Distributions from the baseline and ayahuasca conditions were subtracted to compute 95% confidence intervals (CIs) of the difference distributions and *p*-values (the proportion of the distribution greater than 0). Because recollection estimates are zero-inflated when the incidence of high-confidence false alarms is high (the first point of the ROC curve is pulled away from the *y*-intercept), the DPSD model cannot accommodate false memory effects unless one assumes impaired recollection. Contrary to this assumption, false memory manipulations that produce such reductions in recollection estimates are not accompanied by reductions in hit rates (Doss et al., 2018b, 2023b) or performance on recollection-based tasks (e.g., verbal free recall, Ranganathan et al., 2017). Thus, the DPSD analysis only contained targets and unrelated lures.

The HMeta-d Toolbox for Matlab was used for hierarchical Bayesian meta-*d*′ modeling and is discussed in detail elsewhere (Fleming, 2017). Briefly, the probability of a correct response (i.e., *p*[“old”|target + “new”|lure]) is plotted against the probability of an incorrect response (i.e., *p*[“new”|target + “old”|lure]) in a cumulative fashion starting with the highest level of confidence to the lowest level of confidence with the final point always being (1,1). A ROC curve is then fit to these data. However, such “type 2” ROC curves scale with overall memory (i.e., type 1) performance. That is, the metacognitive measure is confounded with how good one’s memory is when one should be able to have a good memory and not know this about themselves or a bad memory and know this about themselves. Because each type 1 *d*′ (i.e., *z*[hit rate] − *z*[false alarm rate]) has a “best case” type 2 ROC curve if one were to have perfect correspondence between their type 1 and type 2 performance, a type 1 *d*′ can be interpolated from the type 2 ROC curve produced by the actual data that assumes such perfect correspondence between type 1 and type 2 performance. This interpolated type 1 *d*′, termed meta-*d*′, is in the same units of the *d*′ produced from the actual data, thereby allowing type 1 performance to be taken into account. The ratio between meta-*d*′ and *d*′ is termed metacognitive efficiency and is referred to here as “metamemory.”

For each drug condition, we used default settings of the HMeta-d toolbox, specifically 3 Markov Chain Monte Carlo chains, discarding the first 1000 samples of each chain to allow for model convergence followed by 10,000 samples. Like the recollection and familiarity estimates, distributions from the baseline and ayahuasca conditions can be subtracted to compute a 95% CI and *p*-value. Due to the multiple types of targets and lures, we explored several methods for estimating metamemory including all targets (non-suggested and suggested) and all lures (suggested, related, and unrelated), targets and unrelated lures, targets and related lures, and suggested targets and suggested lures.

## Results

### Memory performance

**Figure 1.**
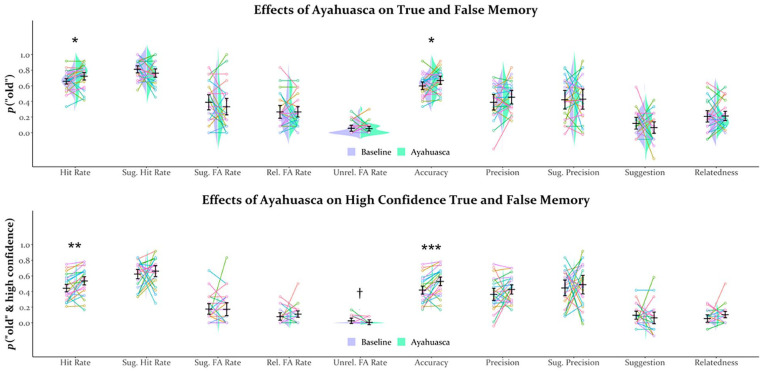
Split-stringed violin plots for memory performance including all levels of confidence (a) and only high-confidence responses (b). Horizontal bars reflect the mean, error bars reflect the 95% confidence interval, and “strings” reflect individual participant data with consistent colors for a given participant. *p < 0.050. **p < 0.005. ***p < 0.001. ^†^*p* < 0.100.

Table 2 and Figure 1 display the results of the analysis of memory performance. Familiarity ratings for scenes during encoding did not significantly differ between drug conditions (CI = [−7.11 to 9.91], *t*(23) = 0.34, *p* > 0.250), suggesting that participants visually explored scenes similarly sober and on ayahuasca. As shown in Figure 1(a) and (b), pre-encoding ayahuasca administration (further referred to as *pre-encoding ayahuasca*) surprisingly enhanced memory, as evidenced by increased hit rates (CI = [0.01–0.12], *t*(23) = 2.44, *p* = 0.023, *d* = 0.50), memory accuracy (CI = [0.01–0.13], *t*(23) = 2.30, *p* = 0.031, *d* = 0.47), and especially, high-confidence hit rates (CI = [0.04–0.15], *t*(23) = 3.34, *p* = 0.003, *d* = 0.68) and high-confidence accuracy (CI = [0.05–0.17], *t*(23) = 3.90, *p* < 0.001, *d* = 0.80). Further evidence for a memory enhancement from pre-encoding ayahuasca came from a near-significant reduction in high-confidence unrelated lure false alarm rates (CI = [−0.00 to 0.04], *t*(23) = 1.82, *p* = 0.081, *d* = 0.37; Figure 1(b)), though these were near floor. Pre-encoding ayahuasca did not impact other measures, including measures of memory distortion (Table 2 and Figure 1).

**Table 2.** Memory Performance.

Dependent Variable	Mean ± SEM or SD [95% CI]			Baseline vs. ayahuasca			
	Baseline	Ayahuasca	Δ	*t*(23)	*p*	*d*	BF
Scene familiarity rating	70.26 ± 4.03 [61.93, 78.60]	68.86 ± 3.27 [62.10, 75.62]	1.40 ± 4.11, [−7.11, 9.91]	0.34	0.736	0.07	4.42
All confidence responses							
Hit rate	0.66 ± 0.03 [0.61, 0.71]	0.72 ± 0.03 [0.66, 0.78]	0.06 ± 0.03 [0.01, 0.12]	2.44	0.023	0.50	2.43
Sug. Hit Rate	0.81 ± 0.03 [0.76, 0.86]	0.76 ± 0.03 [0.70, 0.83]	0.05 ± 0.04, [−0.03, 0.13]	1.28	0.214	0.26	2.26
Sug. FA Rate	0.39 ± 0.05 [0.28, 0.50]	0.33 ± 0.06 [0.21, 0.45]	0.06 ± 0.05 [−0.05, 0.16]	1.13	0.272	0.23	2.64
Rel. FA Rate	0.27 ± 0.04 [0.18, 0.36]	0.27 ± 0.04 [0.19, 0.35]	0.00 ± 0.04 [−0.08, 0.08]	0.00	1.000	0.00	4.66
Unrel. FA Rate	0.06 ± 0.02 [0.02, 0.09]	0.05 ± 0.01 [0.02, 0.08]	0.01 ± 0.02 [−0.03, 0.05]	0.28	0.780	0.06	4.49
Accuracy	0.60 ± 0.03 [0.54, 0.66]	0.67 ± 0.03 [0.61, 0.73]	0.07 ± 0.03 [0.01, 0.13]	2.30	0.031	0.47	1.92
Precision	0.39 ± 0.04 [0.30, 0.48]	0.45 ± 0.04 [0.38, 0.53]	0.06 ± 0.04 [−0.03, 0.16]	1.47	0.155	0.30	1.81
Sug. Precision	0.42 ± 0.05 [0.32, 0.53]	0.43 ± 0.05 [0.32, 0.54]	0.01 ± 0.06 [−0.12, 0.14]	0.11	0.914	0.02	4.63
Suggestion	0.12 ± 0.03 [0.05, 0.19]	0.07 ± 0.04 [−0.01, 0.14]	0.06 ± 0.06 [−0.06, 0.17]	0.97	0.344	0.20	3.07
Relatedness	0.21 ± 0.04 [0.13, 0.29]	0.21 ± 0.03 [0.14, 0.28]	0.01 ± 0.04 [−0.08, 0.09]	0.13	0.894	0.03	4.62
High-Confidence Responses							
Hit Rate	0.44 ± 0.03 [0.38, 0.51]	0.54 ± 0.04 [0.46, 0.61]	0.09 ± 0.03 [0.04, 0.15]	3.34	0.003	0.68	14.11
Sug. Hit Rate	0.62 ± 0.03 [0.55, 0.69]	0.66 ± 0.04 [0.59, 0.74]	0.04 ± 0.05 [−0.07, 0.15]	0.70	0.489	0.14	3.73
Sug. FA Rate	0.18 ± 0.03 [0.10, 0.25]	0.17 ± 0.04 [0.08, 0.27]	0.00 ± 0.04 [−0.09, 0.09]	0.08	0.938	0.02	4.65
Rel. FA Rate	0.08 ± 0.02 [0.04, 0.12]	0.11 ± 0.02 [0.06, 0.16]	0.03 ± 0.03 [−0.03, 0.09]	1.09	0.288	0.22	2.75
Unrel. FA Rate	0.03 ± 0.01 [0.01, 0.05]	0.01 ± 0.00 [0.00, 0.02]	0.02 ± 0.01 [−0.00, 0.04]	1.82	0.081	0.37	1.12
Accuracy	0.42 ± 0.03 [0.35, 0.49]	0.53 ± 0.04 [0.46, 0.60]	0.11 ± 0.03 [0.05, 0.17]	3.90	0.001	0.80	46.72
Precision	0.36 ± 0.04 [0.28, 0.45]	0.43 ± 0.03 [0.36, 0.49]	0.06 ± 0.04 [−0.03, 0.15]	1.42	0.168	0.29	1.92
Sug. Precision	0.45 ± 0.05 [0.35, 0.54]	0.49 ± 0.05 [0.38, 0.60]	0.04 ± 0.08 [−0.12, 0.21]	0.52	0.609	0.11	4.12
Suggestion	0.10 ± 0.03 [0.04, 0.15]	0.06 ± 0.04 [−0.01, 0.14]	0.03 ± 0.04 [−0.06, 0.12]	0.80	0.432	0.16	3.49
Relatedness	0.05 ± 0.02 [0.01, 0.09]	0.10 ± 0.02 [0.06, 0.15]	0.05 ± 0.03 [−0.01, 0.11]	1.69	0.105	0.34	1.36
DPSD model							
Recollection	0.29 ± 0.07 [0.12, 0.40]	0.49 ± 0.05 [0.40, 0.57]	0.20 ± 0.08 [0.08, 0.34]	—	0.000	—	—
Familiarity	1.27 ± 0.11 [1.06, 1.46]	1.20 ± 0.09 [1.03, 1.35]	0.07 ± 0.14 [−0.21, 0.30]	—	0.326	—	—
Meta-*d*′ Model							
Metamemory All	0.75 ± 0.08 [0.60, 0.88]	0.69 ± 0.09 [0.52, 0.83]	0.07 ± 0.12 [−0.17, 0.26]	—	0.281	—	—
Metamemory Targ.-Unrel. Lure	0.67 ± 0.11 [0.46, 0.85]	0.82 ± 0.09 [0.63, 0.97]	0.15 ± 0.14 [−0.13, 0.38]	—	0.148	—	—
Metamemory Targ.-Rel. Lure	0.79 ± 0.13 [0.55, 1.00]	0.88 ± 0.13 [0.62, 1.10]	0.08 ± 0.19 [−0.28, 0.39]	—	0.328	—	—
Metamemory Sug. Targ.-Sug. Lure	0.70 ± 0.14 [0.43, 0.93]	0.59 ± 0.15 [0.30, 0.84]	0.12 ± 0.20 [−0.29, 0.45]	—	0.275	—	—

SEM = for memory performance, SD = for DPSD and meta-*d*′ analyses, Hit Rate = *p*(“yes”|target), Sug. Hit Rate = *p*(“yes”|sug. target), Sug. FA Rate = *p(*“yes”|sug. lure), Rel. FA Rate = *p*(“yes”|sug. lure), Unrel. FA Rate = *p*(“yes”|unrel. lure), Accuracy = Hit Rate − Unrel. FA Rate, Precision: Hit Rate − Rel. FA Rate, Sug. Precision = Sug. Hit Rate − Sug. FA Rate, Effect of Suggestion (Suggestion) = Sug. FA Rate − Rel. FA Rate, Relatedness Effect (Relatedness) = Rel. FA Rate − Unrel. FA Rate, Recollection = DPSD recollection estimate, Familiarity = DPSD familiarity estimate, Metamemory = metacognitive efficiency (meta-*d*′/*d*′), Metamemory All = metamemory using targets + suggested targets and suggested lures + related lures + unrelated lures, Metamemory Targ.-Unrel. Lure = metamemory using targets and unrelated lures, Metamemory Rel. = metamemory using targets and related lures, Metamemory Sug. Targ-Sug. Lure = metamemory using suggested targets and suggested lures.

BF: Bayes factor; CI: confidence interval; DPSD: dual process signal detection; FA: false alarm; SD: standard deviation; SEM: standard error of the mean.

### DPSD and meta-*d*′ modeling

**Figure 2.**
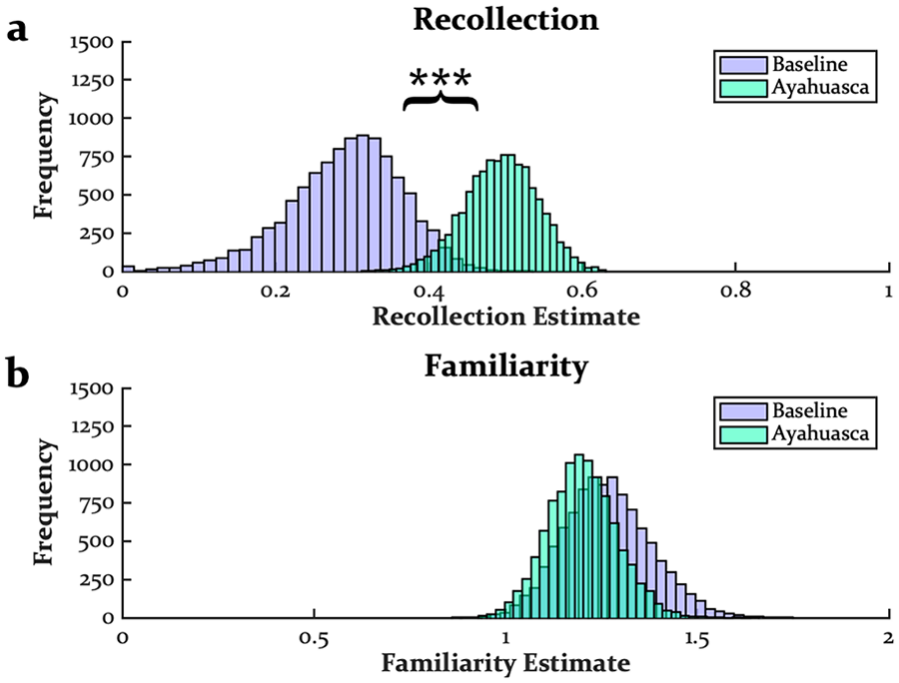
Distributions of recollection (a) and familiarity (b) estimates from DPSD modeling. DPSD: dual process signal detection. ***p < 0.001.

Figure 2 displays the distributions of recollection and familiarity estimates from the DPSD analysis (see also Table 2). As would be expected from an increase in high-confidence hit rates without an increase in high-confidence false alarm rates, recollection estimates were enhanced by ayahuasca (*M* = 0.20, SD = 0.08, CI = [0.08–0.34], *p* < 0.001; Figure 2(a)). Although pre-encoding ayahuasca did not impact familiarity estimates (*M* = 0.07, SD = 0.14, CI = [−0.21 to 0.30], *p* > 0.250; Figure 2(b)), we note that familiarity estimates at baseline and under ayahuasca were rather elevated compared to past work, a point we return to in the Discussion.

**Figure 3.**
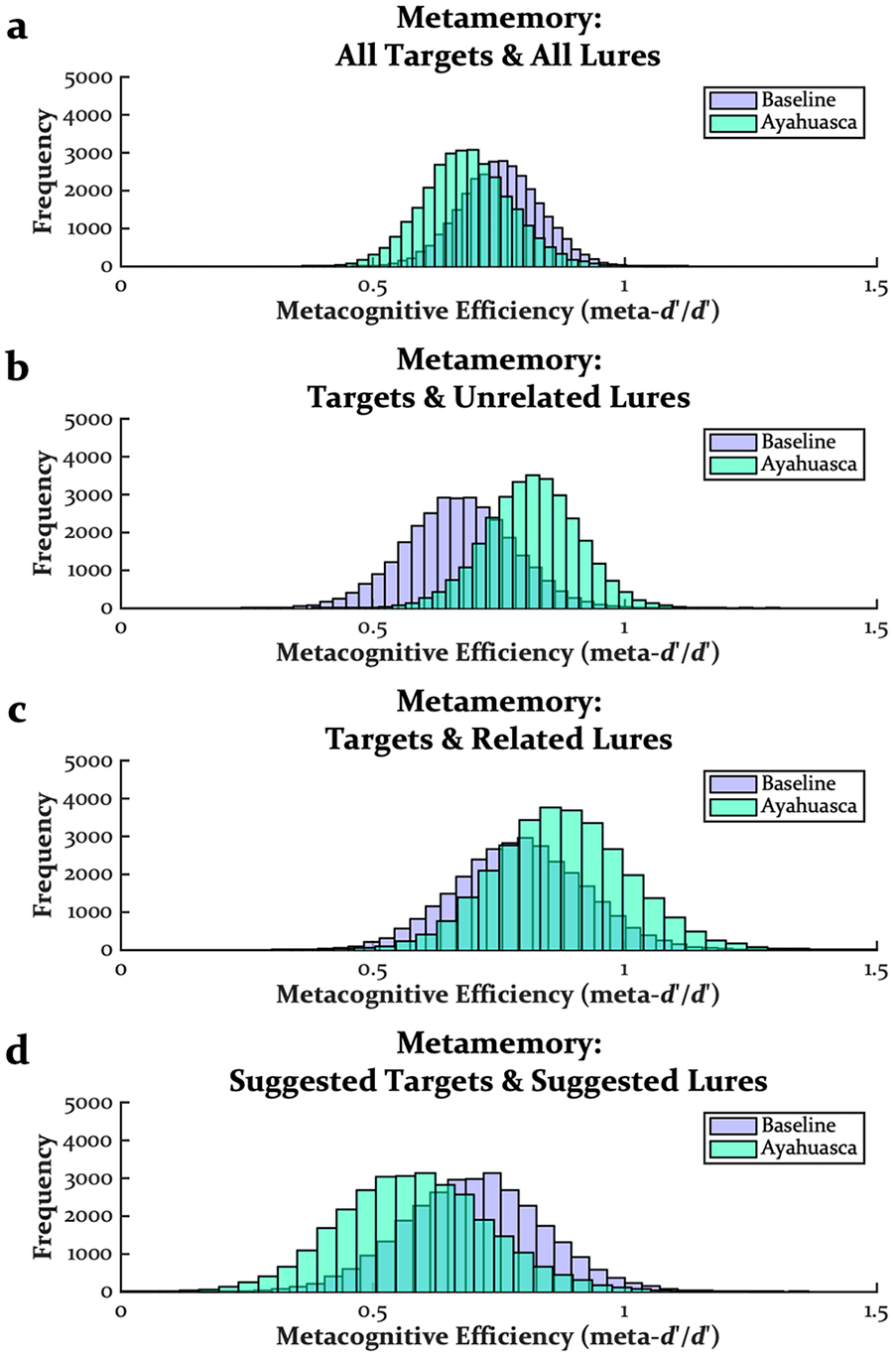
Distributions of metamemory estimates from meta-*d*′ modeling of all targets and all lures (a), targets and unrelated lures (b), targets and related lures (c), and suggested targets and suggested lures (d).

Figure 3 displays the distributions of metamemory estimates from the meta-*d*′ analyses (see also Table 2). Pre-encoding ayahuasca did not impact metamemory using all targets and all lures (*M* = 0.07, SD = 0.12, CI = [−0.17 to 0.26], *p* > 0.250; Figure 3(a)), targets and unrelated lures (*M* = 0.15, SD = 0.14, CI = [−0.13 to 0.38], *p* = 0.148; Figure 3(b)), targets and related lures (*M* = 0.08, SD = 0.19, CI = [−0.28 to 0.39], *p* > 0.250; Figure 3(c)), and suggested targets and suggested lures (*M* = 0.12, SD = 0.20, CI = [−0.29 to 0.45], *p* > 0.250; Figure 3(d)). Thus, regardless of how metamemory was estimated, there was no evidence that pre-encoding ayahuasca impacted metamemory, consistent with the effects of psilocybin and MDMA at encoding or retrieval (Doss et al., 2023b).

## Discussion

We found that pre-encoding ayahuasca in experienced ayahuasca users of the Santo Daime church surprisingly enhanced memory, specifically by increasing hit rates, accuracy, high-confidence hit rates, high-confidence accuracy, and DPSD recollection estimates, but had no impact on DPSD familiarity estimates, which were rather elevated at baseline in comparison to previous work (Doss et al., 2023b). Also contrary to expectations, pre-encoding ayahuasca did not increase susceptibility to false memory formation via suggestion or relatedness. Finally, despite the acute effects of ayahuasca being present during retrieval, there was no evidence that ayahuasca increased false memories to unrelated lures, as found with several psychoactive drugs during retrieval (Doss et al., 2023a). Despite not correcting for multiple comparisons across these three types of false alarms, there was no evidence that ayahuasca distorted memory in experienced ayahuasca users.

Why ayahuasca might enhance the formation of recollection-based memories in experienced users is perplexing considering that pre-encoding psilocybin and MDMA typically impair memory (Doss et al., 2023b), and experienced psychedelic users typically exhibit less cognitive impairment than non-psychedelic users, not enhancement, during acute psychedelic effects (Bouso et al., 2013; Healy, 2021). Moreover, hippocampal and entorhinal 5-HT_2A_ receptors are mostly expressed on inhibitory neurons (Bombardi, 2012; de Filippo et al., 2021; Deng and Lei, 2008; Lüttgen et al., 2004; Shen and Andrade, 1998; Wyskiel and Andrade, 2016). Although a single dose of psilocybin downregulates 5-HT_2A_ receptors in both the cortex and hippocampus (Raval et al., 2021), one possibility is that frequent ayahuasca use causes greater downregulation of inhibitory hippocampal 5-HT_2A_ receptors that would otherwise attenuate recollection. Nevertheless, it remains unclear why recollection was enhanced rather than not impaired by pre-encoding ayahuasca. One clue may come from the doses of DMT and β-carbolines. Whereas 170.64 mg of β-carbolines was similar to prior work, 3.36 mg of DMT was 10–20 times lower (Callaway et al., 1999; Dos Santos et al., 2011, 2012; Riba et al., 2003). Despite this low dose of DMT, plasma concentrations of DMT and subjective effects (see Ramaekers et al., 2023) were similar to those from studies administering ayahuasca containing higher DMT doses. Thus, one possibility is that DMT does in fact enhance the encoding of recollection-based memory in experienced users who purportedly exhibit “reverse tolerance,” which would allow even low doses of DMT to impact cognition. Alternatively, MAO-A inhibition via β-carbolines could have driven the recollection enhancement (cf. Allain et al., 1992; Wesnes et al., 1989), and some work has found that harmine specifically can enhance memory in animals (Dos Santos and Hallak, 2017). Perhaps even the low dose of DMT contributed to this effect, though microdoses do not typically enhance cognition (Bershad et al., 2019; Family et al., 2019; but see Hutten et al., 2020; Yanakieva et al., 2019 for studies with selective enhancements in which caffeine was not permitted to be consumed). If β-carbolines can truly enhance memory encoding, their coadministration in psychedelic therapy may be warranted to counteract the memory impairments otherwise produced by psychedelics. Nevertheless, given the negative correlations between plasma harmine and improvements in high confidence hit rates (the strongest relationship across all correlations, see Supplemental Material but interpret with caution), there may be diminishing returns or even memory impairments with higher doses of β-carbolines. Future work should explore the possibility that β-carbolines could attenuate the psychedelic-induced impairments of forming hippocampal-dependent memories.

Another observation was that baseline familiarity estimates in this study were rather high, especially considering the use of a task that taxes recollection. Prior work has found experienced ayahuasca users to have better executive functioning (Bouso et al., 2012) and verbal free recall (Grob et al., 1996) compared to non-using populations, though one study found worse verbal free recall in ayahuasca-using versus non-using adolescents (Doering-Silveira et al., 2005). Although it is difficult to assess the significance of the elevated familiarity in the present study, familiarity estimates were ≈25% greater than a reanalysis of several datasets containing cued recollection tasks (Doss et al., 2023b). Across 40+ placebo and drug manipulations in this reanalysis, familiarity enhancements produced by pre-encoding psilocybin and MDMA were of the few cases that rivaled the elevated familiarity estimates of the present study. Note that enhanced familiarity can increase false memory formation (Doss et al., 2016, 2020a), though the enhancement of recollection may have precluded such effects. A possible explanation for elevated familiarity may be cortical plasticity (Calder and Hasler, 2023; Inserra et al., 2021; Olson, 2018). Familiarity is thought to be an aggregate signal of perceptual and semantic processing (Johnston et al., 1985; Ozubko and Yonelinas, 2014; Wang and Yonelinas, 2012), and a greater number of connections in the cortical processing hierarchy could allow for more cortical spread during encoding, thereby enhancing subsequent familiarity.

This study had several limitations such as the observational study design that lacked a placebo condition and control group of infrequent users. Furthermore, because the ayahuasca session always took place after the baseline session, the recollection enhancement under ayahuasca could be due to practice effects. However, while some degree of practice effects was likely present, we believe that practice effects are unlikely to completely account for the recollection enhancement for a few reasons. First, we attempted to minimize practice effects by implementing a practice version of the task prior to the baseline assessment and tested memory for different stimuli that were counterbalanced between the two sessions. Second, the recollection enhancements from pre-encoding ayahuasca were large enough such that a power analysis of this effect on high-confidence accuracy (α = 0.05, power = 0.80, *d* = 0.80) suggested that it could have been detectable with only 15 participants. The enhancement of recollection was also larger than any drug-mediated enhancement of recollection in a reanalysis of 10 datasets (Doss et al., 2023b). This reanalysis also contained a dataset in which memory was tested on the same task 16 times with the effects of zolpidem occurring on tests 6–8 and 10–12, yet the recollection enhancements between the 1st and 16th test were much smaller than those reported here (cf. Kleykamp et al., 2012). Furthermore, zolpidem’s amnestic effects were large enough to overcome any practice effects, whereas the large memory enhancements in the present study would suggest that even if ayahuasca impaired the encoding of hippocampal-dependent memory, which is typically a reliable effect of prototypical psychedelics and MDMA in humans and animals (Barrett et al., 2018; Doss et al., 2023b; Kloft et al., 2022; Rambousek et al., 2014; van Wel et al., 2011; Zhang et al., 2017), such an impairment could not have been very robust. If there were general practice effects, then it begs the question why only recollection was impacted but not other measures on this task. For example, participants did not become better at avoiding false memories nor did any measure of metamemory improve (even without corrections for multiple comparisons), which might be expected if participants had learned how to perform the task better. Moreover, performance on other cognitive tasks was not impacted across sessions (Ramaekers et al., 2023). The memory enhancements from pre-encoding ayahuasca were also larger than any of the improvements in executive functioning on a range of cognitive tasks in a study with a similar design (i.e., baseline followed by acute ayahuasca in experienced users, Bouso et al., 2013). Nevertheless, some evidence suggests that episodic memory can be more susceptible to practice effects than executive functioning (Glisky et al., 2022). Given the inflated optimism in psychedelic research (Appiani and Caroff, 2024; Doss et al., 2022; Yaden et al., 2022), controlled trials are needed to validate this potential encoding enhancement.

Another limitation was that ayahuasca was active across encoding, consolidation, and retrieval. Considering that there was little delay between encoding and retrieval, drug effects on consolidation were likely minimized (but see Doss et al., 2023a; Doss et al., 2018c for how GABAA sedatives can retroactively enhance memory with relatively short encoding-retrieval delays). Furthermore, no drug to our knowledge enhances retrieval (i.e., when drug effects are isolated to retrieval), and when psychoactive drug effects have been isolated to retrieval, including the effects of MDMA, the only effect has been an increase in false memories (Doss et al., 2023a). Finally, because ayahuasca enhanced recollection, it could have also enhanced memory for the misinformation. Better memory for misinformation could increase susceptibility to false memories, though such an effect may have been mitigated by the enhancement of accurate recollection. One study also suggests that frequent ayahuasca users may be less susceptible to mnemonic interference (Barbosa et al., 2016). Regardless, the present study would suggest that ayahuasca does not increase susceptibility to false memory when an event takes place and is remembered all under acute effects in frequent ayahuasca users (but note that the findings here cannot generalize to inexperienced populations, and our sample was relatively homogenous). Considering that ayahuasca is being explored for its therapeutic potential (Palhano-Fontes et al., 2019; Sanches et al., 2016), it will be important to examine whether inexperienced users are also not more susceptible to memory distortion under the acute effects of ayahuasca. Future work with delays between memory phases (for drug clearance) and precisely timed drug administrations (i.e., pre-encoding, immediately post-encoding, pre-misinformation, immediately post-misinformation, and just prior to a delayed memory test) will delineate the temporal dependency of drug effects on encoding, consolidation, retrieval, and memory distortion.

## Supplemental Material

sj-docx-1-jop-10.1177_02698811241301216 – Supplemental material for Ayahuasca enhances the formation of hippocampal-dependent episodic memory without impacting false memory susceptibility in experienced ayahuasca users: An observational study Supplemental material, sj-docx-1-jop-10.1177_02698811241301216 for Ayahuasca enhances the formation of hippocampal-dependent episodic memory without impacting false memory susceptibility in experienced ayahuasca users: An observational study by Manoj K Doss, Lilian Kloft, Natasha L Mason, Pablo Mallaroni, Johannes T Reckweg, Kim van Oorsouw, Nina Tupper, Henry Otgaar and Johannes G Ramaekers in Journal of Psychopharmacology
