# Supplementary material for: Ayahuasca enhances the formation of hippocampal-dependent episodic memory without impacting false memory susceptibility in experienced ayahuasca users: An observational study
Source: J Psychopharmacol. 2024 Nov 29;39(4):339–49. doi: 10.1177/02698811241301216 (PMC11967096; doi:10.1177/02698811241301216)
Supplement: sj-docx-1-jop-10.1177_02698811241301216 – Supplemental material for Ayahuasca enhances the formation of hippocampal-dependent episodic memory without impacting false memory susceptibility in experienced ayahuasca users: An observational study [file sj-docx-1-jop-10.1177_02698811241301216.docx]

**Ayahuasca Enhances the Formation of Hippocampal-Dependent Episodic Memory**

**Without Impacting False Memory Susceptibility in Experienced Ayahuasca Users:**

**An Observational Study**

**Supplementary Material**

Manoj K. Doss^1,2†^*, Lilian Kloft^3†^, Natasha L. Mason^3^, Pablo Mallaroni^3^, Johannes T. Reckweg^3^, Kim van Oorsouw^4^, Nina Tupper^4^, Henry Otgaar^4,5^, & Johannes G. Ramaekers^3^

^1^Department of Psychiatry and Behavioral Sciences, Center for Psychedelic Research & Therapy, The University of Texas at Austin Dell Medical School

^2^Department of Psychiatry and Behavioral Sciences, Center for Psychedelic & Consciousness Research, Johns Hopkins University School of Medicine

^3^Department of Neuropsychology and Psychopharmacology, Faculty of Psychology and Neuroscience, Maastricht University

^4^Department of Clinical Psychological Science, Faculty of Psychology and Neuroscience, Maastricht University

^5^Leuvens Institute of Criminology, Faculty of Law and Criminology, KU Leuven

^†^Equal contributions

*Correspondence to: Manoj K. Doss, 1601 Trinity Street, Bldg. B, Austin, TX 78712

ORCID: 0000-0003-2939-2522

Email: manoj.doss@austin.utexas.edu

Keywords: episodic memory; false memory; recollection; familiarity; psychedelics; ayahuasca

*Exploratory Correlations*

We ran an exploratory correlation analysis between all memory performance measures (see Methods), as well as changes in memory performance measures (ayahuasca - baseline) and measures of drug consumption including the amount of ayahuasca consumed (in mL) and maximum plasma concentrations (ng/mL) of *N*,*N*-dimethyltryptamine (DMT), harmine, harmaline, tetrahydroharmine, and β-carbolines (sum of harmine, harmaline, and tetrahydroharmine; Table 1). Methods for obtaining these plasma concentrations have been previously published (Madrid-Gambin et al., 2022). A plasma sample for one participant was not able to be obtained. The strongest correlation was plasma harmine concentrations and changes in high-confidence hit rates (Figures S1 and S2). This negative correlation was counterintuitive considering that high-confidence hit rates increased under ayahuasca. Therefore, there may be diminishing returns or even memory impairments at higher doses of harmine. Although all correlations between significant memory improvements (hit rate, accuracy, high-confidence hit rate, and high-confidence accuracy) and plasma β-carbolines were likewise negative, these correlations should be interpreted with caution, considering the high number of tests.

**Table S1**

|  | Consumed Ayahuasca | Max. DMT | Max. Harmine | Max. Harmaline | Max. THH | Max. β-carbolines |
| --- | --- | --- | --- | --- | --- | --- |
| Mean  (*SD*) | 24.04  (8.24) | 16.54  (15.04) | 7.83  (7.39) | 1.72  (1.50) | 43.37  (27.14) | 52.93  (34.96) |

Mean consumption of ayahuasca (mL), maximum (Max.) plasma concentration of *N*,*N*-dimethyltryptamine (DMT; ng/mL), harmine (ng/mL), harmaline (ng/mL), tetrahydroharmine (THH; ng/mL), and total β-carbolines (sum of harmine, harmaline, and THH; ng/mL).

**Figure S1**


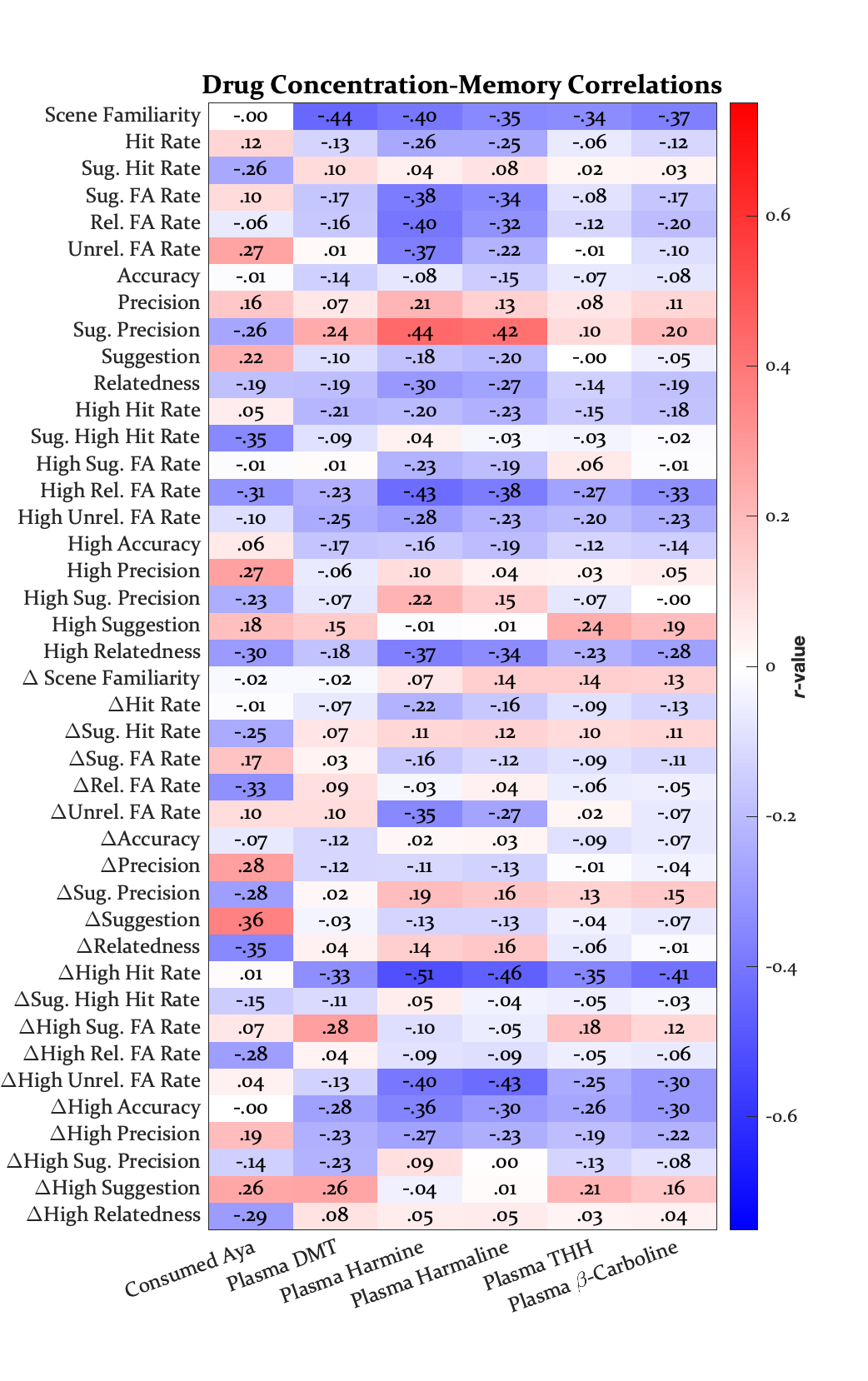


Correlations between consumed ayahuasca (Aya), maximum plasma *N*,*N*-dimethyltryptamine (DMT), maximum plasma harmine, maximum plasma harmaline, maximum plasma tetrahydroharmine (THH), and total maximum plasma β-carbolines (sum of harmine, harmaline, and THH) and all memory performance measures. These correlations should be interpreted with caution, considering the high number of tests. hit rate = *p*(“yes”|target), suggested hit rate = *p*(“yes”|suggested target), suggested lure false alarm rate = *p*(“yes”|suggested lure), related lure false alarm rate = *p*(“yes”|related lure), unrelated lure false alarm rate = *p*(“yes”|unrelated lure), Accuracy = *p*(“yes”|target) - *p*(“yes”|unrelated lure), Precision = *p*(“yes”|target) - *p*(“yes”|related lure), Suggested Precision = *p*(“yes”|suggested target) - *p*(“yes”|suggested lure), Suggestion = *p*(“yes”|suggested lure) - *p*(“yes”|related lure), Relatedness = *p*(“yes”|related lure) - *p*(“yes”|unrelated lure), FA = false alarm, Sug. = suggested, Rel. = related, Unrel. = unrelated, High = high-confidence (only including “yes” responses with the highest level of confidence), Δ = change between ayahuasca and baseline.

**Figure S2**


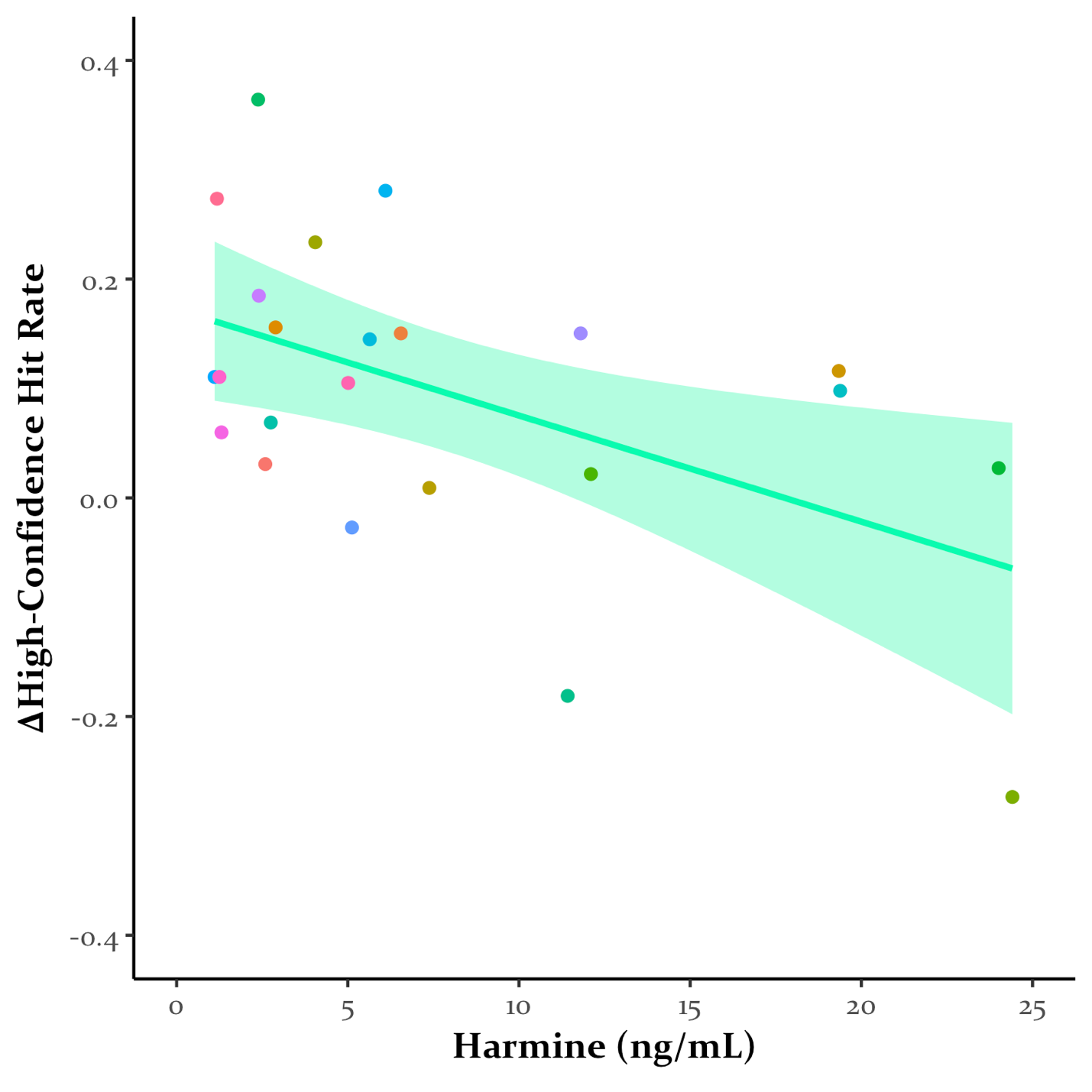


Correlation between change plasma harmine concentrations and change (Δ) in high-confidence hit rates (ayahuasca - baseline). This correlation should be interpreted with caution, considering the high number of tests.

**References**

Madrid-Gambin, F., Gomez-Gomez, A., Busquets-Garcia, A., Haro, N., Marco, S., Mason, N. L., Reckweg, J. T., Mallaroni, P., Kloft, L., Van Oorsouw, K., Toennes, S. W., De La Torre, R., Ramaekers, J. G., & Pozo, O. J. (2022). Metabolomics and integrated network analysis reveal roles of endocannabinoids and large neutral amino acid balance in the ayahuasca experience. *Biomedicine & Pharmacotherapy*, *149*, 112845. https://doi.org/10.1016/j.biopha.2022.112845
